# Supplementary material for: Schedule and magnitude of reproductive investment under immune trade-offs explains sex differences in immunity
Source: Nat Commun. 2018 Oct 22;9:4391. doi: 10.1038/s41467-018-06793-y (PMC6197210; doi:10.1038/s41467-018-06793-y)
Supplement: Supplementary file 1 — Supplementary Information [file 41467_2018_6793_MOESM1_ESM.pdf]

## Supplementary Information

# Schedule and magnitude of reproductive investment under immune tradeoffs explains sex differences in immunity

Metcalf and Graham


| | |
|---|---|
| Supplementary Figures 1 – 7 | p. 2 – 8 |
| Supplementary Note 1 | p. 9 |
| Supplementary Table 1 | p. 10 |

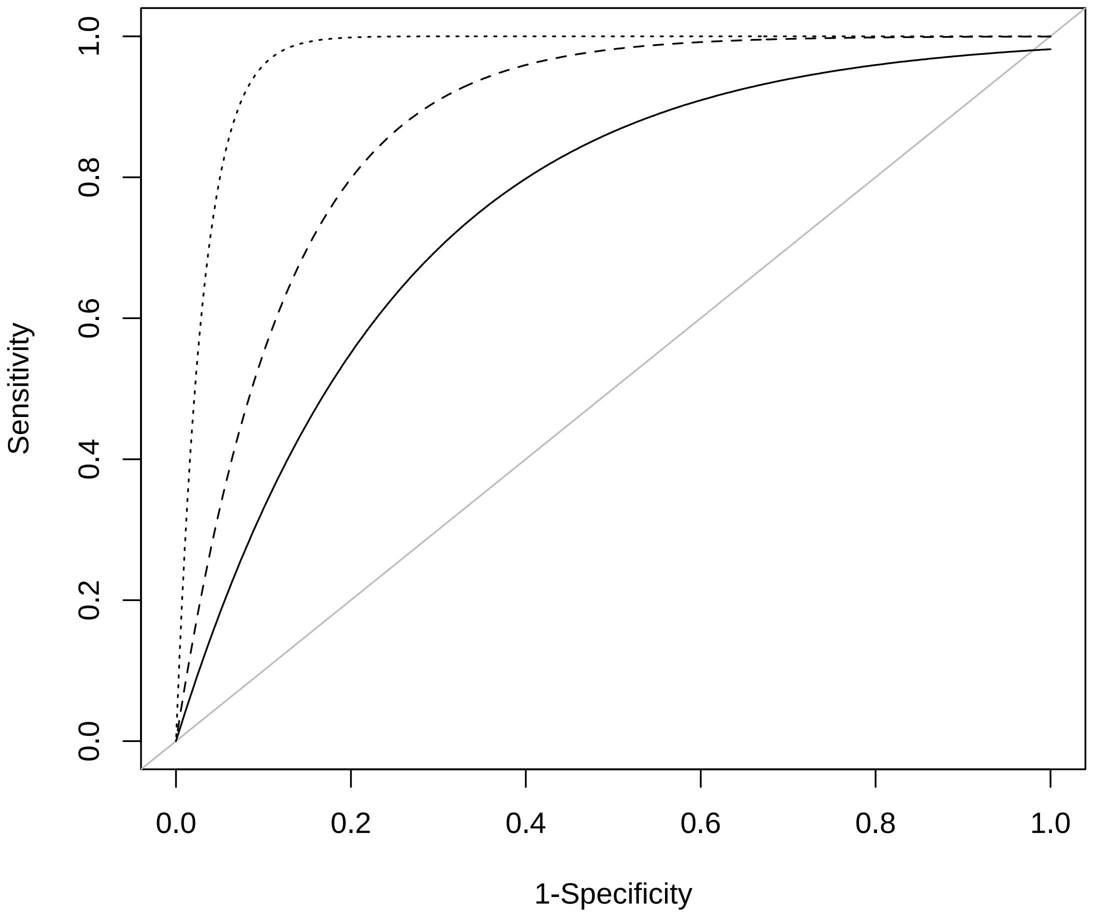


**Supplementary Figure 1: Discrimination trade-off linking sensitivity (y axis) and specificity (x axis,** noting that this is plotted as 1-specificity, in line with the literature from epidemiology). The trade-off between the proportion of 'true' positives detected (sensitivity) and 'true' negatives detected (specificity) emerges as a result of overlap between self and non-self (see Figure 1). Curves that come closest to the top left corner of the plot show highest potential for accurate discrimination, or lowest perceived overlap between the distribution of host and pathogen (degrees of overlap are illustrated along the x axis, Figure 2), resulting in highest sensitivity (close to 1 on the y axis) and specificity (close to 0 on the x axis, and thus specificity close to 1). We capture this trade-off using the functional form $s_e = 1 - \exp^{-\gamma(1-s_p)}$, depicted here for $\gamma$=4 (solid line, little power of discrimination, suggesting considerable overlap between the two distributions) $\gamma$=8 (dashed line) and $\gamma$=32 (dotted line, best discrimination, suggesting well separated distributions). The grey line indicates where x=y, corresponding to the worst possible scenario, i.e., the two distributions are entirely overlapping, and our ability to discriminate self from non-self is only as good as a coin flip. By assuming just one dimension of discrimination (i.e., only one axis of overlap), we constrain the shape of the relationship linking sensitivity and specificity between this x=y line, and a sharply angled line curving through the top left corner. This implies that both the magnitude of sensitivity and the acceleration of gains in sensitivity as a result of reductions in specificity are relatively restricted. Given the importance of the shapes of trade-offs in modulating optimal strategies, it is important to note that more nuanced multivariate distributions could result in more complex shapes, which has the potential to alter the landscape of optimal strategies.

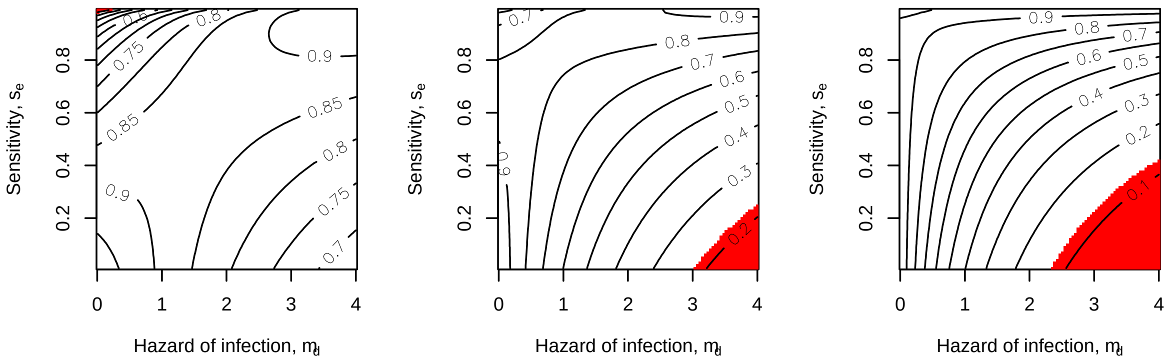


**Supplementary Figure 2: Optimizing survival across a range of scales of infection incidence.** Combining a sensitivity/specificity trade-offs (y axis on each panel) with a trade-off around the magnitude of the immune response (x axis on each panel) yields a bimodal landscape of survival, with high survival corresponding to either low pathogen detection (low sensitivity, $s_e$, y axis) and low pathogen associated mortality ($\mu_d$ , x axis) corresponding to high levels of immune response and thus $\mu_i$; or high pathogen detection (high sensitivity) and high pathogen associated mortality ($\mu_d$ , x axis) resulting from a relatively small magnitude immune response. As infection incidence increases (left to right $i_x$=0.1, 0.5, and 0.9 respectively) the span of parameter space where high sensitivity is optimal increases, and is paired with lower optimal hazard associated with infection.

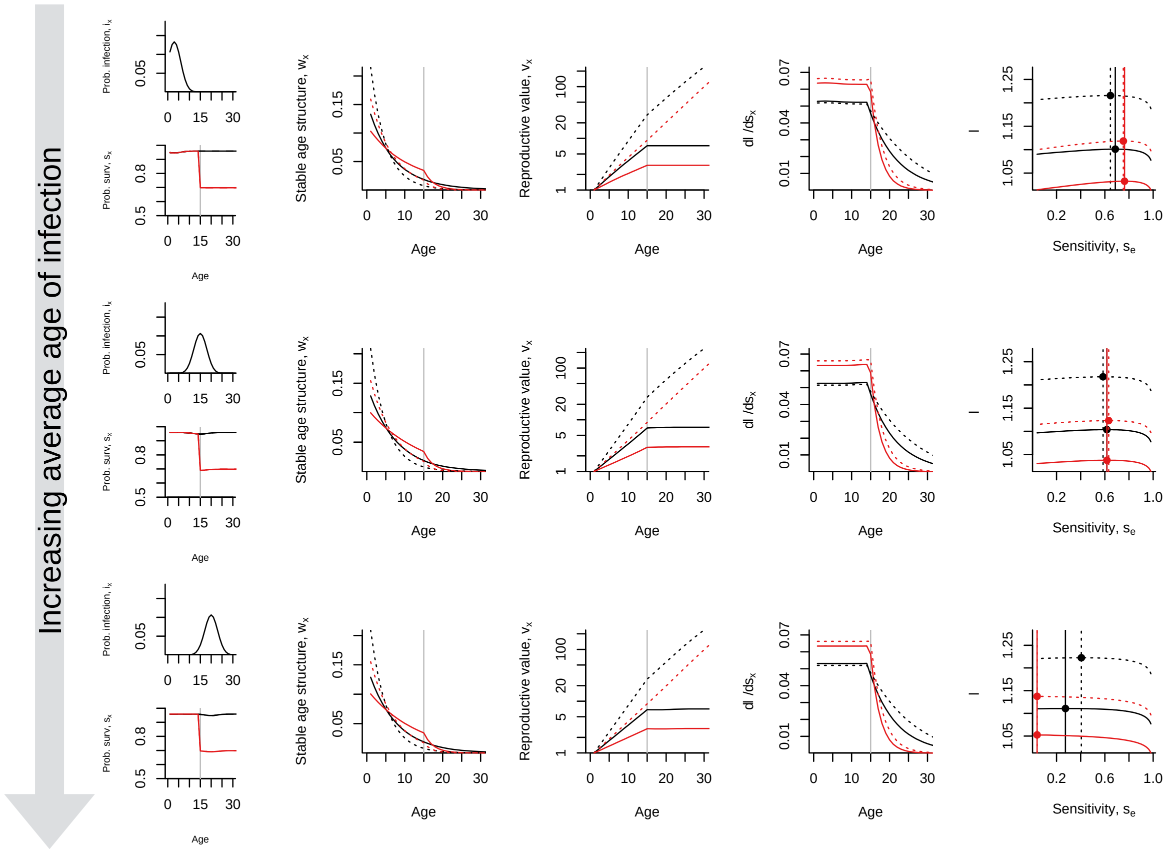


**Supplementary Figure 3: Impact of average age of infection on effect of a change in survival and on fitness.** As the average age of infection (rows) increases (top left panel in each row shows age incidence), probability of survival is affected at later ages (bottom left panel in each row), both for scenarios with no increase in female mortality during reproductive years (black lines) and those with such an increase (red lines) for life histories both without (solid lines) and with (dashed lines) an increase in fertility with age. The age of infection alters the stable stage structure (2nd column) and reproductive value (3rd column) ultimately altering the sensitivity of survival at each age (4th column, y axis), thus changing the optimal sensitivity, $s_e^*$ (5th column) as identified by the value of $s_e$ that maximizes $\lambda$.

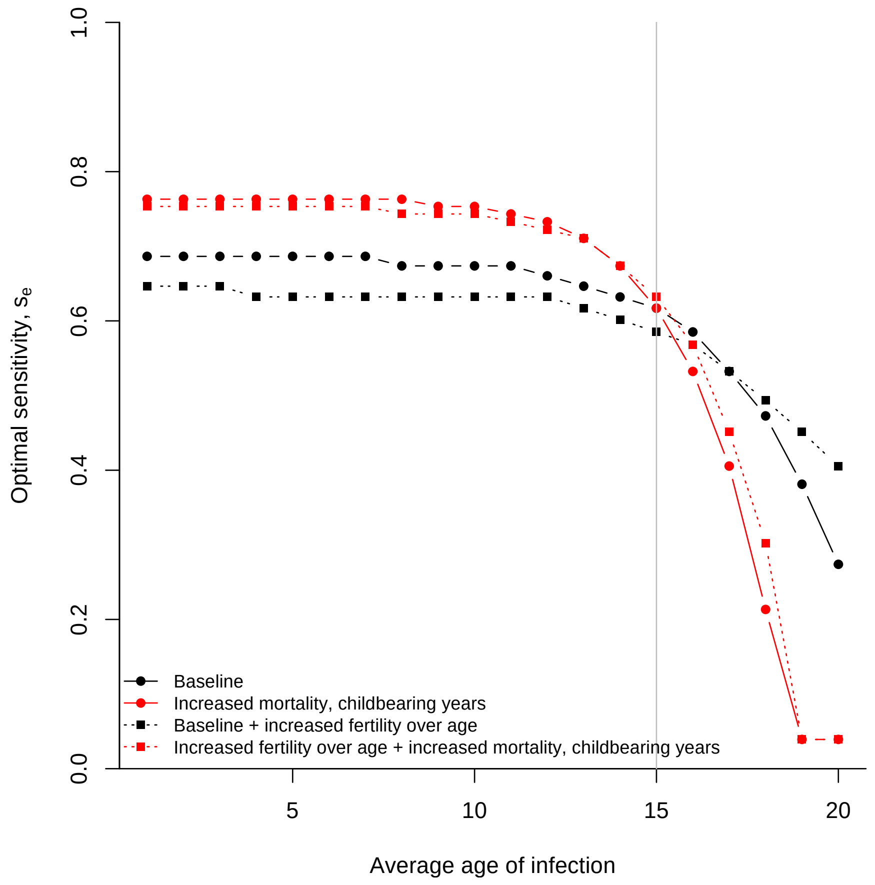


**Supplementary Figure 4: Average age of infection and non-infection related demography** Although $\mu_b$ and fertility do not directly enter the expression for the optimal sensitivity within an age class (see Methods), the ways in which they shift the age structure of infection and the contribution of different ages to fitness can weight changes in $s_e$ relative to changes in the pattern of infection over age. As the average age of infection (x axis) increases, the optimal sensitivity, $s_e^*$ (y axis), decreases, in line with basic evolutionary demography theory. Increasing mortality during reproductive years increases selection for sensitivity for young ages of infection (red circles above red squares, and black circles above black squares for young ages) but this pattern reverses for older ages (black circles and squares below the red circles and squares).

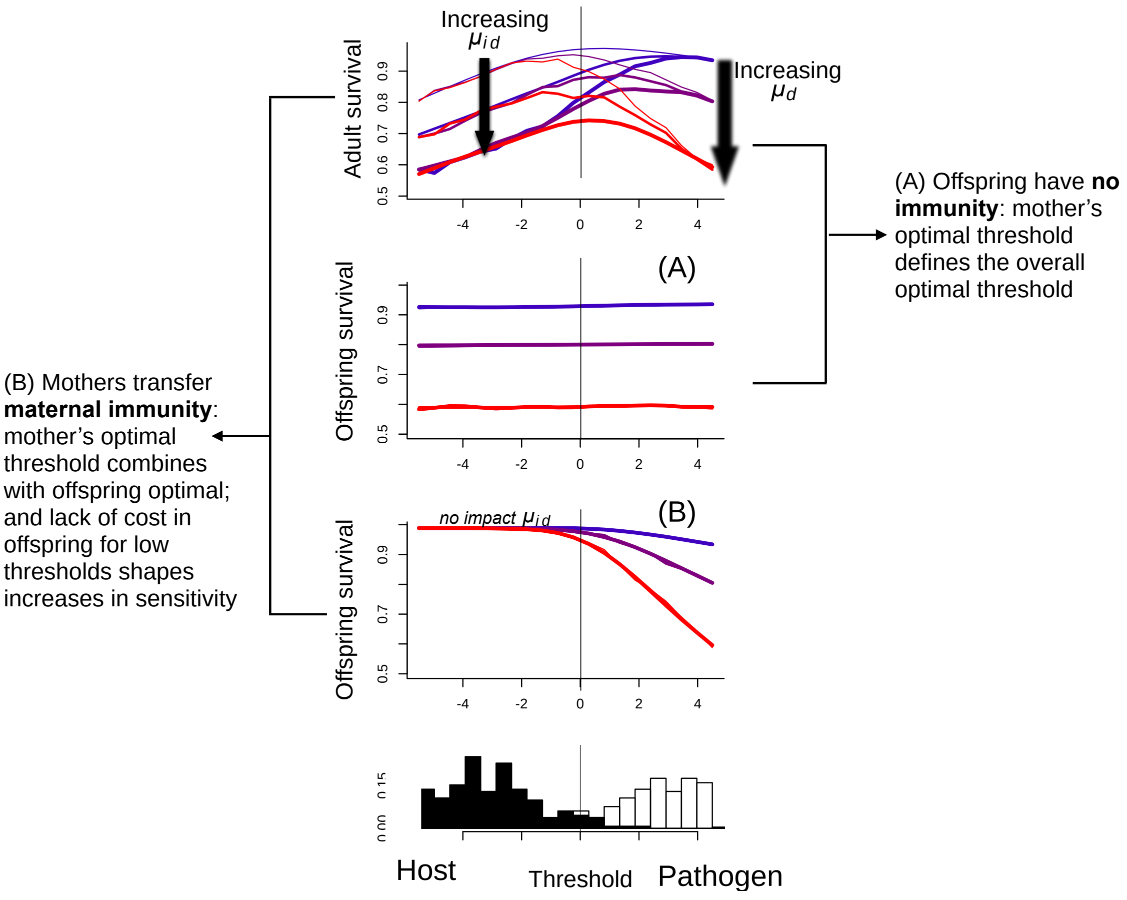


**Supplementary Figure 5: Individual based model simulation showing impact of maternal immunity on sensitivity threshold** (see Supplementary Note 1 for details, Supplementary Data 2 for code)**.** Across a range of values of the threshold defining the discrimination trade-off (*x* axis, high sensitivity to the left, and specificity to the right) the y axis shows survival of adults (top panel) and of offspring (A) without any immunity (maternal or otherwise), and (B) with maternally transferred immunity, for a range of values of $\mu_d$ (increasing from 0.01 (blue line) to 0.1 (red line), right hand arrow, top) and $\mu_{id}$ (increasing from 0.01 to 0.1, reflected in line thickness, left hand arrow, top). Lowest panel shows schematic of host and pathogen distributions across values of the threshold. In adults (top panel), as pathogen induced mortality increases (gradient from blue to red) the threshold that maximizes survival shifts to the left for the same level of immunopathology. As immunopathology induced mortality increases (comparing lines of the same color but increasing thickness), the threshold that maximizes survival shifts to the right. In scenario (A), offspring survival is independent of the threshold, and simply declines with the magnitude of pathogen induced mortality ($\mu_d$); the overall optimal threshold is thus defined by that of adults (top panel). In scenario (B) offspring benefit from mother's threshold during the first year of life (assuming maternal antibodies are transferred), and pay none of the penalty associated with immunopathology ($\mu_{id}$). Evolution will favour the threshold at which both adult and offspring survival is maximized, tilting the optimal to lower thresholds and higher sensitivity in the presence of maternal immunity, especially if offspring are more vulnerable to infection induced mortality. See Supplementary Figures 6-7 for the underpinnings of various optima.

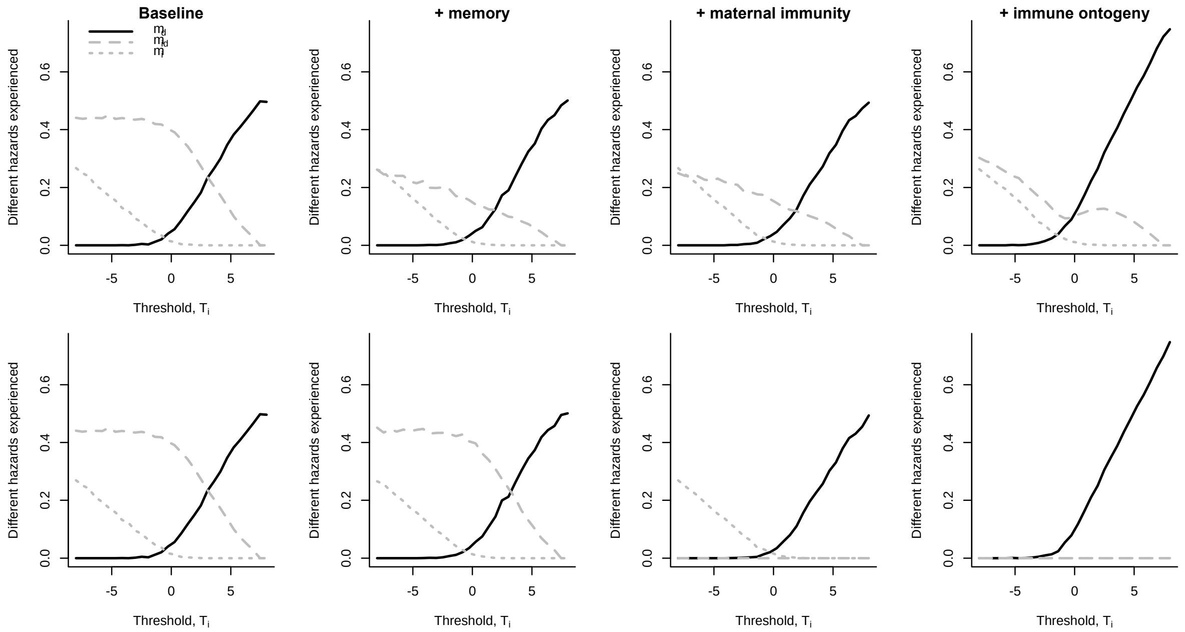


**Supplementary Figure 6: Simulated contributions of different hazards** for mothers (top row) and offspring (bottom row) across a scale of magnitudes of the threshold for immune discrimination, as in the conceptual framing in Figure 4, but now simulating a stochastic set of pathogen epitopes in every year and tracking individuals and their reproduction and offspring survival for scenarios defined by plot titles. All scenarios show an increase in disease-related hazard $\mu_d$ (solid black line), as the threshold $T_i$ increases, and fewer epitopes corresponding to infections are above the threshold; and a reduction in immunopathology-related hazard, $\mu_i$ (dotted grey line) as the threshold increases and fewer epitopes corresponding to 'self' are above the threshold; and finally a reduction in appropriate disease response related hazard, $\mu_{id}$ (dashed grey line) as fewer infections are caught as the threshold moves up. Adding memory (second column) reduces the hazard associated with appropriate reactions to infection in adults ($\mu_{id}$); adding memory and maternal immunity reduces both $\mu_{id}$ completely in offspring (lower row, third column); removing immunity in the youngest individuals (i.e., implementing immune ontogeny) results in much greater immune related disease in the adults (fourth column, top row), and removes all immune related hazards in offspring (fourth column, lower row). The optimal threshold in each case reflects the minimum sum of these different hazards, see Supplementary Figure 7.

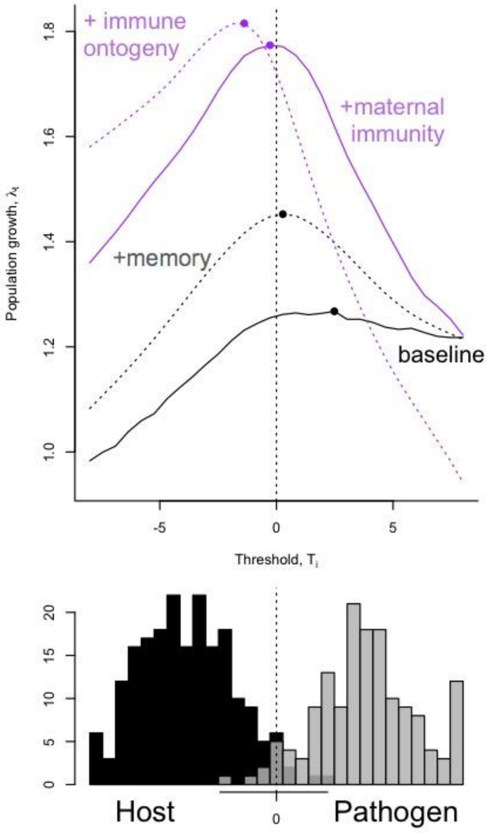


**Supplementary Figure 7: Simulation of different scenarios** showing population growth across the set of combinations illustrated in Figure S6. Individuals face a novel distribution of pathogens every year (illustrated in the lowest panel), and the population either has no memory and no maternal immunity (black), memory only (black dashed line) or memory and maternal immunity (purple), or memory and maternal immunity in a context where offspring have under-developed immunity in the first year of life (purple dashed line). In this setting, each additional layer reduces the threshold, thus increasing optimal sensitivity in females, relative to the baseline, or memory only case, which will reflect the optimal in males. However, the direction of the effect of adding maternal immunity can go in either direction unless the immune system takes a while to develop.

**Supplementary Note 1: Simulating the effects of adaptive immunity and transfer of maternal antibodies**

We simulated a population of host individuals, indexed *i*, each characterized as having a distribution of 'self' epitopes normally distributed along a continuous axis. Every year, hosts are subjected to a distribution of pathogens reflected by 'non-self' epitopes, again taken as being normally distributed. Every host individual has a threshold $T_i$ defining the level above which an epitope is classified as 'non-self'. For each host, each pathogen falling above this threshold incurs a mortality hazard of $\mu_{di}$, each pathogen falling below this threshold incurs a mortality hazard of $\mu_d$; and each 'self' epitope falling above this threshold incurs a mortality hazard of $\mu_i$, in addition to the baseline mortality hazard to which each individual is exposed, $\mu_b$, thus directly reflecting the analytical model developed above.

Our model focusses on the female part of the population, and every year, surviving mothers may also produce one offspring. The offspring's epitopes are taken to be distributed normally with a defined variance, and a mean defined as the average of the mother's epitopes and a randomly drawn variable from the distribution of epitopes across the population, to implicitly reflect the father's contribution. The offspring's threshold *T* is set to reflect the mother's threshold. Every epitope of the offspring that is above the mother's threshold $T_i$ incurs a small probability of miscarriage (which can be used to illustrate results shown in Figure 3H). If this does not occur, the offspring's survival probability relative to that year's distribution of pathogen epitopes is calculated as above.

To extend this model to encompass adaptive immunity, we also keep track of a 'memory' of epitopes for each time-step. In simulations encompassing memory, after the first exposure, the value of memory reflecting that epitope changes from 0 to 1, and exposure to pathogens reflecting epitope no longer result in any excess mortality (either $\mu_d$ or $\mu_{di}$). To additionally capture transfer of maternal antibodies, we afford the protection from their mother's memory to offspring to survive their first year of life; after this phase, they revert to having a memory set to 0 for all epitopes. Finally, to reflect the fact that the immune system may still be under development in the first year of life, we evaluate the impact of young individuals being affected by $\mu_d$ whatever the epitope value of the pathogen and their own threshold, but conversely, experiencing none of the damage associated with the immune response (thus not incurring hazards $\mu_{di}$ or $\mu_i$).

For tractability, we discretized the underlying continuous variable of epitope space taken as spanning the arbitrary range of values from -10 to 10 into 30 bins. To prevent extinction, if the number of surviving individuals fell to 0, we sampled 20 individuals at random from individuals in that time-step to continue the population. To maintain a maximum population size *N*, if the number of surviving individuals exceeded this value, we sampled a number of individuals reflecting this excess at random and removed them from the population. The parameters of the model are defined in Supplementary Table 1. We also tested the model to ensure that there was no sensitivity to starting conditions, and that output aligned with expectations. Code is available in the file Supplementary Data 2.

**Supplementary Table 1: Model parameters in the Individual Based Model**, description, and, where appropriate, defining relationships characterizing the core trade-offs under investigation.

| Symbol | Description | Details |
|---|---|---|
| $\boldsymbol{H_i}$ | A vector of epitope values for each host, reflecting the number of 'self' epitopes in each of *n* bins across a continuous scale. | Generated for each individual from a normal distribution (with mean set to -5 for the starting population, and defined as the average of the mother's distribution, and the distribution in the population for offspring generated during the simulation). Subsequently discretized into *n*=30 even bins across the focal epitope space range (here -10 to 10). |
| $\boldsymbol{P_i}$ | A vector of pathogen epitope values to which hosts are exposed each year, reflecting the number of 'non-self' epitopes in each of *n* bins across a continuous scale. | Generated from a normal distribution in each year with mean of, e.g., +5; and subsequently discretized as above. |
| $\boldsymbol{M_i}$ | A vector of memory for epitope values for each year, generated by the host, in discrete bins as above. | Initiated at 0 for all epitope levels, and then turned to 1 at subsequent exposure. |
| $T_i$ | Host threshold for discrimination | Unless the corresponding value of memory is 1, pathogen epitopes above this level incur $\mu_{di}$ (reflecting damage associated with the immune response), and below this level incur $\mu_d$ (damage associated with failure to detect a pathogen); host epitopes above this level incur $\mu_i$. see Table 1.<br>Offspring epitopes above this level each incur a probability of miscarriage $c_i$. |
| $c_i$ | Probability of miscarriage associated with each offspring epitope above the mother's threshold. | Impacts not investigated in detail here; potentially an important driver of the evolution of plasticity in mammalian female immunity. |
