# Supplementary material for: Schedule and magnitude of reproductive investment under immune trade-offs explains sex differences in immunity
Source: Nat Commun. 2018 Oct 22;9:4391. doi: 10.1038/s41467-018-06793-y (PMC6197210; doi:10.1038/s41467-018-06793-y)
Supplement: Supplementary file 3 — Description of Additional Supplementary Files [file 41467_2018_6793_MOESM3_ESM.pdf]

Description of Additional Supplementary Files

File Name: Supplementary Data 1
Description: R Code used to generate analyses and figures

File Name: Supplementary Data 2
Description: Code reflecting an individual based model to test the impact of maternal immunity
